# Evaluating Female Experiences of Electronic Dating Violence in Jordan: Motivations, Consequences, and Coping Strategies

**DOI:** 10.3389/fpsyg.2021.719702

**Published:** 2021-11-30

**Authors:** Rula Odeh Alsawalqa

**Affiliations:** Department of Sociology, The University of Jordan, Amman, Jordan

**Keywords:** bullying prevention, cyber abuse, cyberbullying, dating violence, digital dating abuse (DDA), intimate partner violence, violence

## Abstract

Gender stereotypes can influence electronic dating violence (EDV) because the victims’ experiences with abusers depict crucial social mechanisms concerning relational dependency and unequal power relations between men and women, making it difficult for women to resist, report, or escape cyber abuse. In the Arab context, cyber abuse in romantic relationships has not been sufficiently examined. This study investigated female experiences of EDV through a qualitative exploratory descriptive approach. Participants experienced several short- and long-term negative psychological and emotional behavioral responses. Our findings validate that EDV heightened the probability of intimate partner violence definitively via psychological, emotional, verbal, and physical abuse. Their resistance strategies differed according to the extent and nature of the abuse. None of the participants sought help from family due to fear of being killed or forced out of university, and realizing that they would continue to experience multiple forms of abuse. Rather, they either sought help from female professors at the university or paid the abuser to be left alone. Further, they engaged in protective behaviors to block their abusive partner’s access to them, consulted an Information Technology expert, and secretly requested assistance from the police. Preference for controlling and dominant roles, gaining monetary benefits, sexual exploitation, peer pressure, and revenge and anger due to abandonment were the leading motivations for abuse. Female students in their first year of university, those who lived in a disjointed family environment, or those who suffered abuse from their families were particularly susceptible to being victimized. Moreover, passwords shared with others or accounts left open on others’ devices also enabled EDV. Hence, universities must conduct awareness sessions, for female students, on how to manage emotions and safe communication on social media and build healthy friendships and relationships. Curricula, seminars, workshops, and courses in the Jordanian educational sector should include programs and interventions that challenge perceived gender norms. These results have significant practical and clinical implications that help understand EDV in a poorly understood context and provide the groundwork for further research on the EDV problem in Jordan, addressing a lacuna in the literature on violence against Jordanian women.

## Introduction

Given the role of social media in identity construction and psychosocial development, the youth are predominantly present on social media platforms, such as Facebook, WhatsApp, Twitter, Snapchat, and Instagram. These enable them to make more friends, experiment with diverse styles (i.e., emotional, behaviors, and interactional), learn essential social and technical skills, and positively impact loneliness, intimacy, and relationship maintenance (Adams and Bukowski, 2007; Wood et al., 2016; Common Sense Media, 2021). The properties of social media websites allow for the disclosure of personal information via profiles and posts while facilitating the social sharing of emotions (Bazarova et al., 2015; Ostendorf et al., 2020) to receive more likes, gratifying comments, and social and emotional support (Bazarova et al., 2015). However, self-and emotional disclosure through social media may negatively affect the youth and cause numerous privacy and relational issues while harming sensitive aspects of their personal life. They may become exposed to cyberstalking, be vulnerable to peer pressure, face sexual harassment, experience negative mood symptoms, or develop mental disorders (O’Keeffe et al., 2011; Wang et al., 2011; Barlett et al., 2014; Bazarova et al., 2015; Lin and Utz, 2015; Wood et al., 2016; Ostendorf et al., 2020). Besides, they may also encounter several negative emotional effects of social media addiction, such as fear of missing out (FOMO; Bloemen and De Coninck, 2020), burnout and emotional exhaustion (Han, 2018; Liu and Ma, 2020), and Facebook depression (O’Keeffe et al., 2011; Jelenchick et al., 2013), defined as “depression that develops when preteens and teens spend a great deal of time on social media sites, such as Facebook, and then begin to exhibit classic symptoms of depression” (O’Keeffe et al., 2011, p. 802). According to The Child Mind Institute, “teenage and young adult users who spend the most time on Instagram, Facebook and other platforms were shown to have a substantially (13–66%) higher rate of reported depression than those who spent the least time” (Miller, 2021).

Romantic relationships and dating are the most salient and attractive topics during adolescence and young adulthood (Foshee and Reyes, 2011; Lansford, 2011). Such relationships are described in terms of closeness, yearning for intimacy, and emotionality and portrayed in the popular media as endless love, selfless care, and devotion (Shulman et al., 2011). Romantic relationships play a vital and positive role in adolescent and young adult development, building conflict negotiation skills and experiences (Exner-Cortens, 2018) and contributing toward positive adjustment and increased levels of perceived support (Hancock et al., 2017; Ellis and Dumas, 2018). Therefore, abuse faced in romantic relationships and dating negatively affects a person’s physical and mental health, holistic well-being (Chen et al., 2009; Taquette and Monteiro, 2019), and self-worth (Rill et al., 2009). Moreover, for many adolescents who are generally unable to regulate their response, romantic relationships are characterized by heightened emotionality and volatility (Exner-Cortens, 2018). This inability can increase the difficulty of interpersonal interaction in romantic relationships, particularly for those having lower levels of social skills or lacking strategies to deal with stress and frustration (Capaldi et al., 2018). A risky context is thereby created, given the elective and willing nature of the affiliation and the heightened level of emotions concerning romantic relationships (Shulman et al., 2011). The intensity of risks concerning romantic relationships is increasing due to the constant access between romantic partners through digital media (Reed et al., 2017).

Electronic dating violence (EDV), also known as digital dating abuse (DDA) or cyber dating abuse, is a serious and pressing social problem for the youth and can be considered an offline abuse tactic (Reed et al., 2017). Hinduja and Patchin (2020, p. 1) conclude that EDV is “a term used to describe physical, sexual, or psychological/emotional violence that occurs between romantic partners through the use of texting, social media, and related online mediums.” EDV–a form of cyberbullying (Hinduja and Patchin, 2011)–occurs specifically between amorous and sexual partners or ex-partners (Flach and Deslandes, 2017); those engaged in this violence exploit social media as a tool for unhealthy and abusive dating behaviors (Reed et al., 2017). Through social media, EDV facilitates several negative behaviors that cause harm to a romantic partner, such as threatening or posting something online to embarrass them, forcing sexual behavior, and monitoring, controlling, and spreading lewd photos (Hinduja and Patchin, 2011; Hancock et al., 2017; Reed et al., 2017). However, explicit intentions and repeated abusive behaviors are not necessary preconditions in this case because harm can be caused the first time, such as by threats of physical harm via electronic messages and use of force to acquire sexual videos or photos (Reed et al., 2017). Rodríguez Domínguez et al. (2020) signified that the levels and intensities of psychological and emotional effects of EDV could serve as better indicators of its severity and consequences rather than the number of occurrences.

Both male and female youth are at risk of experiencing EDV, and its consequences are detrimental. Among university students, EDV victimization was correlated with mental health symptoms, including depression, anxiety, and distress. Moreover, EDV victims experience higher levels of hostility and stress, uncertainty regarding relationships, feelings of loneliness, antisocial behaviors (Bennett et al., 2011; Hinduja and Patchin, 2011; Weingarten et al., 2018; Galende et al., 2020), and sexual coercion (Zweig et al., 2013b). Furthermore, EDV predicted psychological and physical violence among sexual partners during personal encounters (Brem et al., 2015; Marganski and Melander, 2018). Despite the contradictory findings of previous studies regarding prevalence rates and consequences concerning EDV across both genders (Stonard et al., 2014), the consequences of EDV victimization are more detrimental for women (Zweig et al., 2013b; Dick et al., 2014; Reed et al., 2017). For instance, female undergraduate students in the Midwestern United States reported more negative emotional responses (e.g., fear, embarrassment, and negative hypothetical reactions) to sexual messaging than men (Reed et al., 2016).

### Cybercrimes Against Women in Jordan

In 2010, the Jordanian legislature authority temporarily ratified the Information Systems Crimes Law No. 30. Articles 5, 8, and 9 of the law outlined crimes that women, girls, and children may encounter together with a layout of perpetrators’ offenses and punishment sentences. Offenses include intentional photography, interception, or eavesdropping of materials sent via the Internet related to women, girls, and children. The law punishes whoever sends audible, written, or visual materials containing juvenile pornographic or sexual exploitation acts. Further, the law addresses those who intentionally use the Internet to prepare, store, process, display, print, publish, or promote pornographic/criminal activities aimed at the underaged (i.e., less than 18 years of age) or mentally and psychologically disabled persons. The same applies to cases of underage crime incitement (SIGI-Jordan, 2013). In 2015, the Jordanian legislature authority ratified cybercrime Law No. 27. In 2017, the law was amended to include extended protection for people, especially girls and women (SIGI-Jordan, 2017). Additionally, the Public Security Directorate (2021) established the Cybercrime Department in 2008 and expanded it in 2015 under the name “Anti-Cyber Crime Unit.”

The Sisterhood is Global Institute (SIGI-Jordan, 2013, 2020) indicates that gender-based cybercrime, also known as electronic violence against women (eVAW), includes stalking, extortion, sexual harassment, surveillance, spying on computers, and the unlawful use of technology and the Internet. The illicit usage of the Internet entails the forging of images and videos to threaten and harass others, human trafficking for unlawful sexual transactions, impersonation of well-known social public figures, and identity theft to deceive women and underage girls, especially in chat rooms. Most women and girls have neither the understanding nor the tools to deal with cyber-attacks or pursue the perpetrators. The institute states that cyber violence and harassing remarks are a threat to almost 2.7 million female Internet users in Jordan, including one million underage girls. The SIGI-Jordan (2021) indicates that cybercrimes provide entry points for all kinds of sexual crimes against women, girls, and children. Harassment of women, girls, and children entails receiving frequent and unwanted cellular calls, inappropriate text messages or sexual images, exploitation of personal photos used as personal threats, sexual phrases in comments on online posts, and hacking emails and electronic accounts.

In 2017, the Jordanian Ministry of Communications and Information Technology, in cooperation with the Jordanian Department of Statistics, launched a project to uncover the Internet and communication spread in the country. The results of the project titled “Survey of the use and prevalence of telecommunications and information technology in homes 2017” revealed that 97% of Jordanians rely mainly on cellphones to communicate, among which 91% use WhatsApp to socialize and 87.6% interact through Facebook. The survey also reported that 47% of girls and 53% of boys aged 5 or more use the Internet without parental supervision (SIGI-Jordan, 2020).

The Justice Center for Legal Aid (2021) stated that it regularly dealt with girls who were subject to financial and sexual extortion by young men they met on social media. Typically, these men employed scandals as a technique to abuse underage girls. The Center highlighted that the Jordanian culture of social fear, along with the family and societal values, functions to block any judicial prosecution. Besides, the information provided by the Cybercrime Unit, affiliated with the Criminal Investigation Directorate of the Jordanian Public Security Directorate, confirmed a noticeable yearly increase in cybercrime. The unit administrators believe that the number of unreported crimes is relatively high and emphasized that victims’ families either prefer not to report such incidences or lack legal protection.

The Jordanian society is a civil society, where Sunni Muslims comprise the majority. However, the Bedouin Arab and Islamic cultures are still effective and centered on extended patriarchal family units, thereby playing a central role in shaping the cultural and social contexts. Therefore, romantic relationships and dating before marriage are deemed unacceptable to avoid adultery, social delinquency, and sexual activities and build healthy families. Nevertheless, some groups and individuals believe that it is possible to maintain healthy human relationships and friendships between men and women, and that these do not lead to delinquency or forbidden sexual relations, and are not against romantic relationships and dating before marriage, either publicly or secretly. However, both men and women are punished if they violate social norms, especially related to relationships. The punishment for females usually entails severe repercussions, such as impingement of rights, marriage without consent to preserve family honor, physical or verbal violence, or even death (Euro-Mediterranean Human Rights Monitor, 2020). The Amnesty International (2019) report confirmed that Jordanian women are arbitrarily detained without charge or trial for leaving home without male family members’ permission (absence) or a relationship outside marriage (accuse of zina) and forced to undergo a medical examination to ensure their “virginity.” The use of “virginity tests” by the police in Jordan reinforces the discriminatory idea that male family members have a right to monitor and control women’s sexuality. Additionally, the report documented several cases of unmarried women who became pregnant as a result of rape, who were subsequently imprisoned, forcibly separated from their children, or prevented from registering births, and they will be forcibly subject to state care. Moreover, it indicated that most of these detained women left their homes because of abuse or after their guardian prevented them from marrying a partner of their choice. According to Jordanian law, women under the age of 30 require the consent of their male guardian (father, brother, or uncle) to get married. In 2018, The Jordanian Ministry of Social Development established “Dar Amneh” as a safe house to house women who are detained administratively and in so-called “protective custody.” Amnesty International (2019) considered this house a positive step to have resulted in fewer women being detained.

According to a Human Rights Watch (2001) report, honor crimes, honor killing or shame killing refer to “cases of acts of violence, usually murder, committed by male family members against female family members who are perceived to have brought dishonor upon the family. A woman can be targeted by her family for various reasons including, refusing to enter an arranged marriage, being the victim of a sexual assault, seeking a divorce—even from an abusive husband—or committing adultery. The mere perception that a woman has acted in a manner to bring “dishonor” to the family is sufficient to trigger an attack.” These include divorce or separation from the spouse, refusal to consent to an arranged marriage, attachment to an unsatisfactory relationship reported by the family, having sex before marriage or outside marriage, being a victim of sexual rape or sexual assault, and wearing inappropriate clothes (obscene/indecent). Unfortunately, social media in Jordan has become a pretext for honor killings. The Jordanian authorities arrested a young man in his twenties who killed his 14-year-old sister for creating a Facebook account (Dilwani, 2020). The General Iftaa’ Department (2018: Fatwa Number: 3258) declared that honor killing is incompatible with the provisions of Islamic Sharia; if a person kills his female relative on the pretext of protecting honor, it is a forbidden act called “Haram.” SIGI-Jordan (2021) confirmed that 12 family murders occurred within 6 months of 2021; it included 13 female victims.

The present study examined the experiences and consequences of EDV victimization among female university students in Jordan. We investigated complaints of participants who requested help in stopping intimate partner cyber abuse, asking them to define each reported EDV behavior and their own coping mechanisms. Additionally, we explored perpetrators’ motives and the consequences of EDV for women. We believe our qualitative study is the first to focus on and describe female victims’ experiences regarding EDV in Jordan. In such a patriarchal system, we assumed that the secrecy of romantic relationships to avoid stigma and social ostracization provides a context for EDV that enables male perpetrators to have more control over female victims.

## Materials and Methods

### Study Design

This study employed a qualitative exploratory descriptive design that effectively allows researchers to fully understand a given phenomenon while enabling participants to contribute to the development of new knowledge. This methodology helps to uncover the details of events or experiences and the people involved in them, which then helps researchers in analyzing and interpreting rarely evaluated phenomena, such as EDV against female undergraduates in the Arabian context (Sandelowski, 2010; Hunter et al., 2018). This study used Interpretative Phenomenological Analysis (IPA), which is an approach to qualitative, phenomenological research and includes psychological, idiographic, and interpretative components (Gill, 2014). IPA uses structured and semi-structured interviews to gather verbal and non-verbal information, analyzed to uncover and describe underlying themes (APA Dictionary of Psychology, 2021). It is a particularly beneficial philosophical methodology for investigating complex, ambiguous, and emotionally laden topics (Smith and Osborn, 2015). IPA provides detailed insight into participants’ personal experiences, interprets meanings of particular experiences and events, and explicates without distortion (Demuth and Mey, 2015).

### Participants

We utilized a purposive sampling technique to select female participants exposed to dating violence from of a public university in Jordan. This is considered the most appropriate sampling strategy for descriptive qualitative research (Hunter et al., 2018). Female students who identified themselves as experiencing multiple forms of abuse and EDV through social media (i.e., WhatsApp or Facebook) and mobile phones were recruited. They participated voluntarily to help spread awareness among women and to learn how to confront and stop cyber abuse. The sample size comprised 104 participants, ensuring the quality of information acquired and allowing the emergence of important categories and subcategories. These allowed the researchers to capture and describe phenomena in various situations (Hunter et al., 2018). The identities of study participants were not disclosed, and all identifying information was removed and replaced with synthetic data. According to participants’ wishes, there was no audio recording of interviews. Participants were given full information in plain language about the study and how data will be used, to facilitate their comprehension of the research, allowing them to make informed decisions about participation, and we obtained their voluntary agreement to participate.

### Data Collection

We collected data using a semi-structured interview guide developed for evidence-based studies on cyberbullying and EDV (Bennett et al., 2011; Hinduja and Patchin, 2011; Zweig et al., 2013b; Dick et al., 2014; Brem et al., 2015; Reed et al., 2017; Marganski and Melander, 2018; Weingarten et al., 2018) and information provided by female students during a study on cyberbullying and violence against women (VAW) (Alrawashdeh, 2020; Alsawalqa, 2021). Participants were interviewed face-to-face or via telephone. Interviews lasted between 30 and 60 min and were transcribed manually. The researcher was attentive to participants’ mental and emotional states throughout the interviews. The voluntary nature of the research and guarantee of participants’ confidentiality and anonymity were emphasized to promote trust. The researcher also used data from a sample of women who previously participated in a 2018–2019 study. They were contacted and re-interviewed to corroborate the accuracy of information used to provide advice to women in similar situations. The new data were collected based on interviews conducted with the remaining participants between November 2020 and February 2021.

RA had a close, respectful relationship with the study participants. She was well-informed about their demographic and personal characteristics. She was also knowledgeable about the participants’ families and understood their customs, traditions, and ways of thinking. This sense of familiarity enabled participants to be more forthcoming and share details of their experiences without much probing. The interviews started with a description of the purpose of the study, and an explanation of how participation was meaningful and important. Easy questions were asked first, in a conversational tone, followed by more open-ended, in-depth questions. Leading questions were avoided. To promote disclosure, RA adopted a non-judgmental attitude, listened intently, showed respect, was empathetic, understanding, and open to the participants’ point of view, and repeated the words used by participants. After each interview, the researcher wrote down her notes and impressions.

### Data Analysis

Following the IPA methodology, this study employed an iterative process refined cyclically for data analysis following thematic analysis. The researcher used secondary coding, besides initial descriptive codes, which were applied to the transcripts line-by-line to enhance the rigor of the study and obtain a deeper understanding of the various constituents and their interconnections. Through an inductive approach, codes were developed by reading the data. After the coding procedure, similar codes were compiled, and the relationships between codes were examined to form major themes (Hunter et al., 2018). The coding system was constructed by reviewing the initial responses and observations and initial and thorough readings of the data, where labels were used for short phrases that represented important and recurring themes in each response. This mechanism enabled us to look for ideas in the text, identify numerous passages of the text that share the same code, and document the observed patterns or themes. A colleague of the researcher (a faculty member in the researcher’s department) cooperated to verify the coding process.

To ensure the credibility of the findings and enhance the validity of the researcher’s interpretations, feedback from the participants regarding the manually transcribed interviews, and the results and quotes included under the study topics, were considered. Qualitative research data were analyzed in terms of the coding process, significant sections selected from the participants’ statements, and the derivation and identification of themes. Analyses were performed per the consolidated criteria for analyzing qualitative research data (Tong et al., 2007) to ensure that participants’ implicit and explicit perspectives and emotions were encompassed and truly representative of the sample. Direct quotes from participants’ narratives were used to support the results.

### Rigor and Reflexivity

The reliability of the interview guide was pretested through initial interviews to check the integrity of the study, formulate clear questions, and conduct discussions in a language devoid of any connotation that may cause embarrassment or tension to the participants. A psychiatrist was consulted regarding the content of the questions and discussion topics to ensure that no harm, such as trauma or exhaustion, was caused to the participants. The data and results were discussed with university academicians involved in conducting research and providing advice about cyber abuse, and an agreement was reached on the reasonableness of the study and its results. All participants were able to speak freely, and their perceptions were accurately represented by the content of the transcribed interviews, which they read and confirmed after each interview (Hunter et al., 2018). Moreover, to reinforce the rigor, criticality, and integrity of the qualitative study, the researcher updated her reflexive diary by recording thoughts, feelings, and personal experiences during the research process. Detailed reflexivity notes/insights on the research process, management of participants’ emotions, and the relationship of the perpetrators with victims were recorded (Palaganas et al., 2017).

## Results

### Sociodemographic Data of Participants

The sample consisted of 104 undergraduate female students, with most participants aged between 18 and 20 years; 69.6% were in the first stage of their undergraduate degree. Participants’ family monthly income ranged from 1060 to 1411.45 US$ (65.3%). About 68% of participants’ fathers and 72.3% of mothers had completed a bachelor’s degree; 96.5% of participants’ parents were still married and lived with each other. The duration of the romantic relationship with abusive partners ranged from 1 to 2 years (89%). Altogether, 98% of the participants reported that the abusive romantic partner was a college fellow, aged between 19 to 22 years, while only a few reported that the abusive romantic partner had attained a postgraduate (6%) or high school degree (4%). Nearly 10% of abusive romantic partners were employed part-time; 90% were unemployed. All participants confirmed that abusive romantic partners used both Facebook and WhatsApp for victimization.

### Themes

According to thematic analysis, EDV against females is defined as aggression or abuse occurring in a romantic relationship between current or former dating partners by threatening, posting, uploading, or sharing sexual or lewd personal photos online, or recording romantic phone calls, video chats, or conversations on social media, as well as sexual and amorous talk to embarrass the victim and blackmail them for money, sexual exploitation, or manipulation. Moreover, four themes emerged from this study that described female victims’ experiences of EDV, including a description of the context of abuse, the perpetrator’s motives and consequences of EDV, coping strategies, and the victims’ perceptions of EDV. The consequences of EDV have been divided into three sections for clarity, wherein the first section describes the negative psychological and emotional effects; the second and third sections describe negative effects related to behavioral responses and intimate partner violence (IPV) associated with EDV, respectively. Table 1 summarizes the themes and their corresponding subthemes.

**TABLE 1:** Summary of the themes.

Theme	Subthemes	Participant Quotes
Motivations for EDV	Anger	*… He was incredibly angry because I agreed to marry my relative, whom my family forced me to marry…* [FV 18].
	Revenge due to abandonment	… *I abandoned him against my will*… [FV 19].
	Monetary gain	*… I did not know that afterward, I would have to give 500 JD to get my photos back…* [FV 11].
	Sexual exploitation	*… I was just a sexual goal, nothing less or nothing more…* [FV 22].
	Enjoying the extremely controlling and dominant role	… *He made me his maid… [FV 4].*
Consequences of EDV	Psychological and emotional effects	… *I am feeling burned out. Now, I feel like a warrior coming back from war… [FV 25]. … I wish I could rid myself of the feeling of shame… [FV 34].*
	Behavioral effects	*… I wake up too early and am not able to get back to sleep… [FV 51].*
	Intimate partner violence (IPV)	*… He kicks me…* [FV 1]. *… He tried to kiss me forcibly…* [FV 101].
Coping Strategies	Seek help; female professors at the university, police	… *The police protected me*… [FV 18]. … *My professor deleted everything*… [FV 7].
	Paid money	… *I gave 200 JD in exchange for his silence*… [FV 7].
	Threat	*… I threatened to get him dismissed from the university*… *He stole the exam questions…* [FV 50].
	Technical defense: Consulted an Information Technology (IT) expert	… *An IT expert hacked his account and got rid of everything*… [FV 44].
Victims’ perceptions of EDV	This theme assessed the attitudes of females toward EDV and contexts affecting abusive-supportive attitudes	… *Society protects the male and does not punish him*… [FV 44]. … *Love is permissible for males and forbidden for females.* [FV 50]. … *In romantic relationships, the female is always the offender*… [FV 90]. … *My family is worse than my abusive partner*… [FV 10]. … *My family instilled fear in me*… [FV 18]. … *Females are deficient in reasoning and religion. we are dealt on the basis of this slogan…* [FV 22]

*EDV, electronic dating violence.*

### Theme 1 – Electronic Dating Violence Against Women: Context and Motivations

#### Context

All participants considered that Jordanian women were more likely to be victims of EDV than the men, and cyber abuse was more likely to be serious when the perpetrator was a male. The participants justified that females were considered responsible for dating abuse since they had engaged in an activity considered unacceptable by society. Besides, the prevailing social concept of masculinity gives men the right to dominate and control women. Females are still considered inferior to males and often treated by the men as irrational, naive, emotional, and easy to deceive. Such an environment prevents women from seeking help, especially parents, due to fear of physical punishment, shame, and social stigma.

*“…because I’m a woman, I’m always the weaker side*…” [FV 43].
*“…Our society does not respect women; the priority is always for men…”* [FV 33].
*“…women are the target…there is nothing to frighten men, men do not bring shame, only women…”* [FV 12].
*“…If my family knew that I was talking to male colleagues or participating in a certain university activity with them, I would be harassed and beaten… I would definitely be killed if they knew that I was in love…”* [FV 25].
*“…My brother is younger than me, but he is the one who interferes in my affairs and has control…, He often beats me for insignificant reasons, and when I complain to my father or mother, the response is known to me: ‘He is your brother, he knows what is in your best interest more than you.”’* [FV 13].
“… *Women are ‘deficient in intelligence and religion’; this is their argument in everything*…” [FV 51].
*“… My sister told me that I brought shame and scandal upon the family.”* [FV 20].

More than half of the participants stated that they first became acquainted with the perpetrator as a colleague while studying at the university and got entangled in a love affair, resulting in a closer relationship with the perpetrator because of their need to share problems and gain support. Some participants had suffered in their family lives from neglect, abuse, poverty, or Parental conflict, while some indicated that the relationship was substantiated by the promises of marriage.

“… *He makes me feel safe, loved, and happy… I did not get that from my family…”* [FV 11].
“… *I am sure my family will do nothing… I am a hopeless case…”* [FV 22].
“… *What do you mean by my father? Dr., do not be too optimistic… I do not see a difference between him and my abusive partner…”* [FV 103].
“… *He promised to marry me after we graduate from university together. I built my dreams with him*…” [FV 5].
“… *I do not deny… he was taking care of me… He gave me money and bought clothes… Took care of my health… My family did not do that…”* [FV 43].
*“… I did not realize that he would be worse than my family…”* [FV 80].

The majority of participants raised the topic of love and romantic feelings. They believed that the concept of love is not sacred to males, and if a woman loves, she is more faithful and honest in her feelings than the man, which makes them more likely to be victims of dating abuse. Contrarily, some participants confirmed that some men value love and feelings, but such aspects are not prioritized.

“…*Men always take advantage of women’s affection*…” [FV 103].
“…*Women sanctify love more than men. Love is the first priority in a woman’s life but the priority of a man is work and money…”* [FV 3].
“…*Gentleness, empathy, and sensitivity are female traits. men use this against women*…” [FV 101].
“…*Even if a man truly loved, he would not defend or fight for love like a woman*…” [FV 61].

Few participants reported experiencing cyber abuse from a woman, but it was not as severe or disastrous as in the case of male abuse. A social stereotype propagates jealousy as a basic feature of women, believed to cause problems and violence among females, driving them to slander and falsify the facts. Moreover, a female victim is more capable of defending herself against a female perpetrator than a male perpetrator.

*“…Last year, one of my female friends threatened me, after a dispute between us, with my personal photos in which I wasn’t wearing the hijab; she stole them from my phone…”* [FV 16].
“…*I made my mother see the WhatsApp messages sent by my cousin, in which she threatened me to publish my phone number and my secrets on Facebook… My mother knows that she is jealous of me…”* [FV 22].
“…*Jealousy of women is fatal…”* [FV 51].
“…*She wouldn’t pose as much of a threat as the male; I managed to stop her*…” [FV 30].

#### Motivations

Most participants attributed the perpetrators’ motivation to the need for money through blackmail or sexual exploitation. Some recalled how much the perpetrators enjoyed controlling them and dominating their personal lives, such as restricting their friends and preventing them from speaking to colleagues. The perpetrators controlled the way they dressed, stipulated the times they would enter and leave the university, and forced them to provide services, such as buying food and cigarettes and completing their academic homework. The perpetrators’ desire for revenge due to abandonment or female colleagues’ jealousy toward the victim drove them to trick and trap the victimized girls in an illusion of love. Furthermore, female victims who shared their passwords or left their accounts open on other’s devices were more likely to be exposed to cyber abuse. Notably, the victim’s family circumstances have a critical role within the context of EDV. Poor economic conditions, frequency of domestic violence, exposure to abuse, and loss of confidence in their family’s ability to protect them make the victims feel like “orphans” and disappointed, weak, unaware, easy to deceive, and accepting of the perpetrator’s negative traits. The following quotes illustrate the motivations for victimization:

*“… I loved him so much… I felt safe with him… I thought that he was joking when he asked me for 100 JD… his threats continued to increase… Then he asked for 200 JD. How stupid was I!*…” [FV 11].
*“… Indeed, our relationship was wonderful. For the first time, I felt feminine and respected. He treated me kindly… I did not expect that he would blackmail me with privately recorded calls between us and threaten me by posting them on social media. I was just a sexual goal, nothing less and nothing more…”* [FV 22].
“…*At first, he amicably requested a sexual relationship. After 2 weeks of my repeated refusal, he swore to send my family the romantic and sexual conversations that we had on WhatsApp, and he had pictures and private conversations with my friends on my Facebook account… He was able to access them because I gave him the password…”* [FV 50].
*“Until this moment, I could not imagine that it was deceit… Believe me, I did not do anything to her (female college)… she paid him money to cause me harm… I loved him with all my heart… We had planned to get married…”* [FV 14].
“… *I was honest with him… he was very angry because I agreed to marry my relative, whom my family forced me to marry… He accused me of betraying him and promised to hurt me just like I hurt him…”* [FV 18].
“… *He was the only person who asked about me and cared for me… my family is in constant conflict… my father is very violent…”* [FV 20].
*“Honestly, he bought me a lot of gifts… everything I wished for… I was very deprived… my family does not respect me nor appreciates my needs…”* [FV 21].

### Theme 2 – Consequences of Electronic Dating Violence

Psychological and emotional effects resulting from victimization were the most emerging and frequent themes among the female victims, which one participant described as burnout and another, drowning. These effects coincided with negative behavioral responses, which negatively impacted academic performance, social roles, and societal interactions.

#### Psychological and Emotional Effects

All the victims in this study suffered numerous negative, psychological and emotional effects during and after their abusive relationships had ended. The suffering of some female victims persisted over a long period. Short-term negative effects included anxiety, stress, depression, low self-esteem, embarrassment, fear, and psychological distress, whereas longer-term negative effects included suicidal thoughts, shame, and feelings of isolation. The participants also showed symptoms of emotional burnout. The following quotes exhibit the effects of victimization:

*“… Instead of being perceived as a victim… I was regarded as the perpetrator and blamed. no one will listen to me…”* [FV 81].
“… *I cannot face others, even myself… I feel burned out”* [FV 60].
*“… Fear kept me from thinking… I was totally handcuffed, completely helpless…”* [FV 55].
*“I felt lost… I was so depressed”* [FV 15].
*“… Believe me, I could not read the exam questions as the lines were intertwined… distractions…”* [FV 31].
“… *I am a bad person… I feel guilty*… *I often thought about suicide”* [FV 9].
“… *I feel irritable without any proximate cause”* [FV 5].
*“… My life became intolerable… I was afraid of the scandal*…” [FV 101].
*“Often, I avoided people and stayed alone to avoid criticism or being attacked…”* [FV 67].

#### Behavioral Effects

The real or perceived threats and exposure to stressors resulting from victimization evoked negative behavioral responses among female victims. The previously negative psychological and emotional effects were associated with triggering factors related to eating disorders. Some participants reported that during abuse, they suffered from anorexia and engaged in emotional eating, in which food served as a source of comfort (binge eating disorder). Furthermore, most participants reported low academic achievement, aggressive behavior, unexplained weight loss, heavy smoking, insomnia, transient tachycardia, and attempted suicide. The behavioral effects of victimization are demonstrated by the following quotes:

*“… He was serious, and I saw my shameful picture with another colleague. My heart was beating intensely… I was exhausted from overthinking… I wished for death so much…”* [FV 33].
“… *I lost eleven pounds or more in less than 6 months without knowing the reason*…” [FV 41].
*“… I had difficulty falling asleep*…” [FV 28].
*“… It was hard to stay asleep*… *I bought sleeping pills”* [FV 13].
*“… I wanted everything to end quickly*… *I felt as if I was drowning… I took several medicines to end my life…”* [FV 80].
“… *I am friendly… but I do not know what happened to me. I became crueler and more aggressive with my colleagues and family…”* [FV 100].
“… *It was a very difficult time… constant anxiety and tension… I was complaining about shortness of breath, a sensation of “flopping” in the chest, and I sometimes fainted. ‘Transient tachycardia’: The doctor told me that…”* [FV 92].

#### Intimate Partner Violence

Intimate partner violence (IPV), also called domestic violence or gender-based abuse, is a gendered phenomenon that refers to any behavior within an intimate relationship that causes physical, psychological, or sexual harm to an individual (Alsawalqa et al., 2021, p. 1).

Electronic dating violence victimization can include the following types of IPV behavior via face-to-face and online interactions: monitoring, intimidation, yelling, harsh words, verbal threats, slander, harassment, monitoring, personal insults, and emotional manipulation. All victims in this study experienced some type of *psychological/emotional and verbal abuse* from an abusive partner, including physical violence, resulting from EDV that varied in frequency and severity. These abuses were repetitive and severe. Furthermore, some participants were defamed, ridiculed, blamed, and received negative comments from university colleagues of both the sexes and members of their families. Such abuses were severe because they occurred secretly between the victim and the aggressor, and the victim could not defend or respond due to the fear of repercussions. Furthermore, the abusive partner used physical violence against the victim as a tactic to enable EDV victimization, demonstrating and reinforcing control for the attainment of goals. Most participants specified being exposed to some form of violent acts, including pushing, punching or kicking, throwing objects, spitting, slapping, pulling (i.e., hand, clothing), touching any part of their body without consent, preventing them from leaving, or forcing them to go somewhere. Hence, EDV is a representation of VAW outside the online sphere. These types of abuse increased the negative psychological and emotional effects of EDV victimization and facilitated the negative behavioral responses of the participants. The following quotes describe some of these abusive behaviors.

*“… I had to ask his permission to do anything… he was interfering in my personal life… he was even choosing the color of my clothes…”* [FV 4].
“… *Pulling my shirt… Spitting in my face…”* [FV 88].
*“… He called me a ‘prostitute’…”* [FV 62].
*“… He cursed me and then slapped me”* [FV 8].
*“… He threw books at me…”* [FV 3].
*“… He told a group of my classmates that I am cheap, stupid, and easy to deceive…”* [FV 44].
*“Every day, my sister reminds me of my shame and is sarcastic toward me. Every day, she wants to prove that she is a better person than me…”* [FV 18].
*“… He told his friends that I had sex with him for money, even though no such thing happened…”* [27]
*“… He was preventing me from sitting with my girlfriends at the university*…” [FV 32].
  *“… While I was with my friends in the university café, he deliberately walked by our side and described my underwear for everyone to hear…”* [FV 17].
*“Every day, he sends me WhatsApp pictures of what I sent him and writes: ‘I will create a scandal’…”* [FV 16].
*“… He informed me on Facebook that he wanted money in exchange for his silence, then shared a recorded call between us, sending a voice message telling me: ‘Behold, you have heard the evidence, I am not threatening in vain; do try me by not sending the money.’…”* [FV 70].

### Theme 3 – Coping Strategies

The victims in this study tried to cope with perpetrators’ threats, negative emotional effects, and psychological stress, which caused EDV victimization, through a set of behaviors to preserve their health and psychological and emotional well-being and stop victimization. Participants’ coping strategies varied according to the severity and nature of the abuse. In relationships that did not last for more than 6 months and were devoid of sexual or humiliating photos, the abusive partner relied on WhatsApp or Facebook chats, which some victims were able to remove with the help of male colleagues who had friendly relationships with the perpetrator. The threats that included sexual or insulting images or recorded video conversations were more harmful as the perpetrator was able to tighten his control, forcing some of the participants to submit to sexual exploitation or pay money for silence. Participants who had a strong personality, awareness, and financial security and passed the second stage of their studies at the university did not surrender or succumb to bargaining, showed indifference or neglect, engaged in behaviors to block their partner’s access to them, and tried to respond to threats via male colleagues. Most consulted an IT expert to hack the abusive partners’ accounts on Facebook and delete all of the victim’s personal information. Moreover, they sought assistance from female professors at the university or secretly requested help from the police. The assistance of the police was helpful; they were able to stop the abuser and delete all the pictures and recorded calls. Moreover, the police, being aware of the cultural and social context in Jordan, especially domestic abuse, and the dire consequences if the families of the females found out about their romantic relationships, cooperated secretly and legally to preserve the families’ safety.

To reduce the negative psychological and emotional effects, some participants consumed sedatives and hypnotic pills, consulted a doctor, sought emotional support from close friends, adjusted expectations with the help of their professor, and utilized distractions, such as shopping, exercise, and sleeping, more than usual:

*“… My professor contacted the abusive partner through my Facebook account… she negotiated with him brilliantly… He responded to her and she met with him…”* [FV 18].
*“… Of course, the abusive partner acquiesced in the professor’s decision… On the contrary, he was quiet embarrassed and tried to justify..”.* [FV 20].
*“… The police officer managed to end the nightmare that I lived in for two continuous months”* [FV 34].
*“… I managed to delete my photos which he had… The IT expert did not charge much money…”.* [FV 41].
*“… I knew he was addicted to Captagon (drug)… I threatened to expose him.”* [FV 70].

## Discussion

This study investigated female experiences of EDV in Jordan using a qualitative exploratory descriptive approach. Results revealed the motives of the abusive partners’ controlling and dominant roles as gaining monetary benefits, sexual exploitation, peer pressure, and revenge and anger due to abandonment. Participants’ coping strategies included seeking help from female professors at the university or paying the abuser to be left alone. They also engaged in protective behaviors to block their abuser partners’ access to them, consulted an Information Technology expert, and secretly requested assistance from the police. Furthermore, EDV heightened the probability of IPV definitively via psychological, emotional, verbal, and physical abuse. Finally, the results revealed that domestic violence and passwords shared with others or accounts left open on others’ devices played a role in enabling EDV.

We believe that our results extend beyond EDV and include a wide range of abuses against Jordanian women. EDV is not only related to the Internet and social media but also an amalgamation of individuals’ real-life interactions. The context of EDV is not limited to the framework of the symbols and meanings in digital culture and facilities provided by the Internet and digital media, rather a realistic reflection of society’s culture. VAW in Jordan arises through coercive control and unequal power relations between women and men within a patriarchal society that imposes rigid masculine gender roles. Men are encouraged to show aggression, adopt controlling behaviors, and promote stereotype-based gender discrimination, whereas women are taught acceptance and subservience to men. Such an ideology exposes women to exploitation and abuse in both real and virtual lives (Alsawalqa, 2021).

Offline dating violence is directly linked with endless discrimination against women and various types of intimate partner abuse (Harway et al., 2002; Eshelman and Levendosky, 2012; Garcia-Moreno et al., 2012) and pinpoints the origin of gender violence (Santoro et al., 2018). Zweig et al. (2013a, p. 8) stated that dating violence among teenagers is linked to psychosocial adjustment, mood disorders, depression, and suicidal ideation. These youngsters are also more likely to report alcohol and other drug use, dieting, eating disorders, binge behavior, early maturation, and sexual activity. Moreover, Reyes et al. (2016) suggested that injunctive norms (i.e., acceptance of dating violence) and traditional gender role-based attitudes work synergistically to increase the risk of dating violence perpetration among men. Therefore, the association between EDV behaviors and gender stereotypes, determined in this study, is not surprising, especially those related to control, aggression, and monitoring behaviors (Lara, 2020). The role of gender stereotypes in forming beliefs and behaviors regarding EDV was manifested clearly when the participants reported being exposed to one or more forms of intimate partner abuse, not only via online modalities but also through physical violence related to sexual abuse. These results are consistent with findings in different cultures; however, Zweig et al. (2013b) found that EDV overlapped with other types of violence and abuse, such as psychological, physical, and sexual violence. Reed et al. (2017) discovered that EDV is linked to offline forms of dating violence, and Caridade et al. (2020) identified that EDV and offline dating abuse (ODA) among adolescents and young adults are quite prevalent and correlated. Significant relationships between control, direct aggression EDV, and physical, verbal, and emotional ODA were established. Concerning victimization or perpetration, control and verbal-emotional ODA were the main risk factors of control EDV victimization and the most prevalent type of abuse.

Additionally, Reed et al. (2021) signified that societal beliefs about gender and dating shape the problematic use of digital media in dating relationships, where males expressed greater affinity toward gender-related stereotyping for perpetration. Conversely, Villora et al. (2019a,b) found no statistically significant association between conformance to masculinity and female gender norms with EDV perpetration or victimization among Spanish university students. Additionally, Branson and March (2021) reported that gender is a non-significant predictor of EDV.

Moreover, our findings indicate that women can act as perpetrators in EDV or cyber abuse, and EDV victimization of men by female partners is possible. However, EDV may be more harmful and serious when the perpetrator is male, and women are more likely to experience EDV than men. In the Arab-Islamic cultural context, female experiences of EDV victimization have a diverse range of negative consequences, such as psychological and emotional effects (e.g., feelings of shame, low self-esteem) and behavioral effects (e.g., eating disorders, transient tachycardia, attempted suicides). To our knowledge, our findings concerning the consequences of EDV, such as attempted suicides, transient tachycardia, emotional burnout, and unexplained weight loss, were not mentioned previously in the literature. Although the results of previous studies are conflicting about gender differences in the perpetration and consequences of EDV (Brown et al., 2020), the gender differences may be less evident (Hancock et al., 2017) or have similar rates of EDV frequency, except for sexual coercion (Reed et al., 2017). Women may be differentially affected by EDV victimization and experience higher levels of negative psychological and emotional responses to EDV than men, such as fear, psychological, or emotional distress (Reed et al., 2017; Smith et al., 2018). Notably, Zweig et al. (2013b) found that male youth were significantly more likely to report perpetrating sexual EDV, while female youth reported greater levels of non-sexual EDV perpetration.

These conflicting results may be due to differences in age groups and cultural contexts. Regarding the Arab-Islamic cultural context, the perceptions of female in this study confirmed that Jordan differs greatly from the Western contexts. It is integral to realize that the context of masculinity and femininity is shrouded in complexity, especially in a more patriarchal society, wherein cultural and social norms are founded on the interconnection between Bedouin traditions and Islamic values and standards. This norm is considered a source for organizing relationships between men and women and understanding gender roles. Over time, this entanglement led to the creation of a complex pattern that allowed some of these standards to be addressed according to personal or collective interpretations in a way that benefited men. Hence, a blurred understanding of gender norms was created that was both irrational and illogical. The systematic review by Rodríguez Domínguez et al. (2020), analyzing 30 instruments for the measurement of EDV among young adults and adolescents, revealed that some factors have contributed to the limited understanding of the EDV phenomenon. For instance, most of the related literature related originated in the United States, containing a high conceptual, methodological, and terminological plurality and a reduced number of instruments with sufficient psychometric guarantees. Additionally, there was an under-representation of indicators concerning sexual EDV, particularly among the instruments applied in Spanish samples.

From the perspective of the study participants, our results revealed that the most prominent motives of the abusive male are: enjoying an extremely controlling and dominant role, gaining money or sexual exploitation, jealousy, anger, and revenge due to partner abandonment. Moreover, jealousy, vulnerable narcissism, and secondary psychopathy are significant positive predictors of EDV. Perpetration of EDV may be best attributed to reactive emotional aggression compared to proactive instrumental aggression (Branson and March, 2021). Additionally, our results were inconsistent with the outcomes of Celsi et al. (2021), which indicated that abandonment was not considered a possible predictor of young adults’ perpetration of EDV. Instead, emotional deprivation and childhood experiences (i.e., abuse, neglect, and witnessing IPV) are possible predictors of perpetrated and suffered EDV, whereby more frequent experiences of emotional abuse and physical neglect during childhood were indirectly related to an increased likelihood of perpetrating EDV among both sexes. Furthermore, colleagues or friends of victims and perpetrators also played a role in the perpetration or prevention of EDV. According to Schell (2018), friend presence, belief in the perpetration of EDV norms, and individual cyberbullying-tolerant attitudes predicted the perpetration of EDV. Our findings showed that other factors may contribute toward female EDV, such as sharing passwords or leaving accounts open on other people’s devices, parental abuse, and poverty. Based on the ecological systems theory, Zweig et al. (2013a, p. 9) noted that family characteristics, including witnessing or experiencing domestic violence, family adversity, dysfunction, and lower parental monitoring, predicted IPV during young adulthood and dating violence victimization. However, good relationships with parents are associated with a decreased probability of reporting teen dating violence. Moreover, Lucero et al. (2014) found that password sharing and account access are the most common abusive actions in socially interactive technology use/abuse among dating teens.

Our study also found that the best coping strategies of EDV include seeking help from people the victim knows and trusts and those who are wise, rational, and experienced. Besides, professional and discreet police interventions may contribute to protecting victims and stopping harm. The victim’s family intervention does not seem to be a good idea in the Jordanian cultural context, as the victim will be treated like the perpetrator and can be murdered, dismissed from the university, and subjected to long-term abuse. Threatening the male offender, in the same manner, may increase the negative consequences of EDV, especially if threatened by a male colleague or friend or when the victim resorts to bargaining with money. Furthermore, the victim’s silence due to the fear of shame and social stigma is not considered a positive coping strategy because it will further expose her to more sexual and financial exploitation and physical violence, and the male offender will not be deterred. These results confirm previous findings of the association between gender stereotypes with violence, especially in an Arab-Islamic cultural context. Nevertheless, there must be a change in the norms, traditions, and social values that form negative stereotypes of women and men, as it reinforces the concept of domination and violence and perpetuates gender inequality; although this is not easy and remains an enduring challenge, it is possible. Jewkes et al. (2015, pp. 122–123) confirmed that hegemonic masculinity, which promotes men’s domination over women, can be used in interventions aiming to change practices and beliefs of patriarchal social system and configuration of masculine ideals, and implemented at a social structural level, individual, and group levels to build gender equity and reduce gender-based violence against women. Moreover, several prevention programs have been developed to help at-risk youth and teens develop healthy relationships, prevent dating violence, and reduce aggressive behaviors (Avery-Leaf et al., 1997; Wolfe et al., 2003; Lee and Wong, 2020). Three effective programs of EDV prevention are reported, namely the DARSI program, the Dat-e Adolescence program, and the Brief Intervention Based on an Incremental Theory of Personality (ITP) in the Prevention of Adolescent Dating Violence (Galende et al., 2020). We recommend similar experiments in Arab societies as well.

## Conclusion

In the context of Arabic and Islamic culture, EDV and cyber abuse against females can be considered as extensions of gender-based violence. Relational violence is multidimensional, within the contexts of coercive control and domination, and has negative effects on women’s mental, physical, and psychological health. This study enables families to know the truth of what women may face if they are exposed to abuse by their parents. Furthermore, women should report on all types of abuse and be taught coping mechanisms, especially related to dating violence. They must also learn to manage their emotions, ensure positive social interaction, establish healthy relationships, and learn safe electronic communication. The results of the study showed that a majority of perpetrators were colleagues of victims at the university. Therefore, we recommend operationalizing violence prevention programs and establishing a special section for such cases in the Information Technology Center at the University of Jordan, including IT specialists and female practitioners of sociology and psychology, to help victims of EDV. Moreover, female awareness regarding EDV problems and positive coping strategies should be facilitated. The EDV problem in Jordan needs further research; we believe that this study will provide the groundwork for further research to fill the knowledge gap on violence against Jordanian women. Furthermore, our results have significant practical and clinical implications that provide an understanding of EDV in a poorly understood context and assessment and prevention of EDV victimization.

## Data Availability Statement

The original contributions presented in the study are included in the article/supplementary material, further inquiries can be directed to the corresponding author/s.

## Ethics Statement

The studies involving human participants were reviewed and approved by Institutional Review Board (IRB) of the University of Jordan, the Hashemite Kingdom of Jordan (reference number IRB/19/2021/689). Written informed consent for participation was not required for this study in accordance with the national legislation and the institutional requirements.

## Author Contributions

RA conceptualized and wrote the original draft, chose the methodology, performed the data analysis, reviewed the final version of the work, and got approval for publication.

## Conflict of Interest

The author declares that the research was conducted in the absence of any commercial or financial relationships that could be construed as a potential conflict of interest.

## Publisher’s Note

All claims expressed in this article are solely those of the authors and do not necessarily represent those of their affiliated organizations, or those of the publisher, the editors and the reviewers. Any product that may be evaluated in this article, or claim that may be made by its manufacturer, is not guaranteed or endorsed by the publisher.
